# Psychological effects of traditional Chinese mind-body exercises for low back pain: A protocol for meta-analysis

**DOI:** 10.1097/MD.0000000000025605

**Published:** 2021-06-04

**Authors:** Qian Huang, Xiaonian Lu, Tianxiang He, Juan Du, Peiguo Zhang

**Affiliations:** a Department of Acupunctue and Tuina, Lianyungang TCM Hospital Affiliated to Nanjing University of Chinese Medicine, Lianyungang; b Department of Dermatology, Huashan Hospital, Fudan University; c Yueyang Hospital of Integrated Traditional Chinese and Western Medicine, Shanghai University of Traditional Chinese Medicine, Shanghai; d Department of Pain, Zibo Central Hospital, Zibo, China.

**Keywords:** low back pain, mind-body exercises, psychological effects

## Abstract

**Introduction:**

Several studies reported that traditional Chinese mind-body exercises showed beneficial effects on improving anxiety and depression of patients with low back pain (LBP) in recent years. However, the effects of traditional Chinese mind-body exercises on improving psychological disorders of patients with LBP remain controversial. Most previous reviews only focused on the effects of traditional Chinese mind-body exercises for LBP on pain and dysfunction. Therefore, the present systematic review and meta-analysis will be conducted to evaluate the evidence on psychological effects of traditional Chinese mind-body exercises for LBP.

**Methods and analysis:**

The electronic databases (PubMed, Embase, MEDLINE, Cochrane Central Register of Controlled Trials, Web of Science, China Knowledge Resource Integrated Database, and Wanfang Data) will be searched. The search will include all documents from their inception to February 2021. The Physiotherapy Evidence Database scale will be used for quality assessment of eligible studies. Risk of bias of eligible studies will also be assessed by Cochrane tool. The meta-analysis will be conducted using the Review Manager Version 5.3 software. The Higgins *I*^*2*^ statistic will be performed to examine for heterogeneity. The subgroup analysis will be conducted based on different types of traditional Chinese mind-body exercises, different intervention time, and different outcomes. Quality of evidence will be assessed using the Grades of Recommendation, Assessment, Development and Evaluation.

**Ethics and dissemination:**

No ethical statement will be required for the performance of this review and meta-analysis. The results of this review will be published in an international peer-reviewed journal.

**INPLASY registration number:**

INPLASY202130075.

**Strengths and limitations of this study**

- The current review will focus on psychological effects including anxiety and depression of traditional Chinese mind-body exercises for patients with LBP.
- This systematic review will only include the evidence from randomized controlled trials of traditional Chinese mind-body exercises including Tai Chi, Qigong, Baduanjin, Yijinjing, Liuzijue, and Wuqinxi for LBP.
- The types of traditional Chinese mind-body exercises may affect the pooled analysis of the included studies. Therefore, the subgroup analysis will be conducted based on different traditional Chinese mind-body exercises.

## 1 Introduction

In recent years, low back pain (LBP) has been a main cause for loss of the ability to work, which resulted in a substantial healthcare burden.^[1]^ The prevalence of chronic LBP is up to 23% of the population worldwide.^[2,3]^ Most patients with LBP suffered chronic pain and limited activities in the life. It is estimated that 24% to 80% of LBP sufferers experienced a recurrence within 1 year.^[4]^ Given the high prevalence of recurrent and chronic pain, most patients with LBP experienced anxiety, depression, and poor quality of life. Therefore, the clinicians should pay more attention to interventions that may prevent recurrences and improve psychological disorders.

Therapeutic exercises are common complementary and alternative interventions used by patients with LBP to decrease chronic pain, improve disability, and strong muscular function.^[5]^ The review also reported that therapeutic exercises showed beneficial effects on pain relief, stiffness alleviation, and disability improvement of patients with musculoskeletal diseases.^[5,6]^ Traditional Chinese mind-body exercises are an ancient way of mental and physical activities, including Taiji, Baduanjin, Yijinjing, Liuzijue, Wuqinxi, and so on, which are low-intensity mind-body exercises focusing on coordinating postures, breathing patterns, and meditation. In China, traditional Chinese mind-body exercises have been a popular therapeutic exercise for patients with LBP as a low cost, highly safe, and easy to learn. Taiji may decrease pain and improve function disability for patients with LBP.^[7,8]^ As mind-body exercises, traditional Chinese exercises also showed beneficial effects on psychological well-being. Studies reported that Taiji improve anxiety and depression due to LBP.^[9,10]^ However, the psychological effects of traditional Chinese mind-body exercises for LBP remains controversial.

Therefore, the current systematic review will be to assess the psychological effects of traditional Chinese mind-body exercises in the management of LBP. The primary outcomes will be focused on anxiety and depression of patients with LBP.

## 2 Methods

This systematic review was registered on the international platform of registered systematic review and meta-analysis protocols with the registration number 202130075. The systematic review and meta-analysis will not require ethical approval because there are no data used in the study that are linked to individual patient data. In addition, the results of this review will be published in an international peer-reviewed journal.

### 2.1 Inclusion criteria for study selection

#### 2.1.1 Type of studies

This meta-analysis will only include randomized controlled trials of traditional Chinese mind-body exercises for patients with LBP. Any case reports, observational studies, cross-sectional design studies, animal studies, letters, and reviews will be excluded. The study protocol and conference abstract of randomized controlled trialswill also be excluded, if detailed information and data will not be provided by the corresponding author.

#### 2.1.2 Type of participants

Patients with a diagnosis of LBP following International Statistical Classification of Diseases and Related Health Problems categories: lumbago, lumbosacral segmental/somatic dysfunction, low back strain, spinal instabilities, flatback syndrome, lumbago due to displacement of intervertebral disc, and lumbago with sciatica. There were no limitations on age, gender, or nationality of patients with LBP.

#### 2.1.3 Type of interventions

In this review, any type of traditional Chinese mind-body exercises, including Taiji, Qigong, Baduanjin, Wuqinxi, Liuzijue and Yijinjing, alone will be included. Combination of any type of traditional Chinese mind-body exercises and an intervention compared with the same intervention (such as Taiji plus traction versus traction) will also be included.

The control interventions may be any kind of therapies without traditional Chinese mind-body exercises, including medicine, observation, manual therapy, acupuncture, traction, education, and so on.

#### 2.1.4 Type of outcome measurements

Primary outcome is psychological syndromes including depression (assessing by Self-rating Depression Scale, Center of Epidemiologic Studies-Depression Scale or any other related scales) and anxiety (assessing by Self-rating Anxiety Scale, State Anxiety Inventory or any other related scales). The secondary outcomes will include pain, which is measured by any pain scales, disability (assessing by Roland–Morris Disability Questionnaire, Oswestry Disability Index or any other related scales) and quality of life (assessing by 36-Item Short Form Survey or any other related scales). In addition, any adverse events are also evaluated.

### 2.2 Search strategy

#### 2.2.1 Electronic searches

We will search the following electronic databases, including 5 English databases (PubMed, Embase, MEDLINE, Cochrane Central Register of Controlled Trials, and Web of Science), and 2 Chinese databases (China Knowledge Resource Integrated Database and Wanfang Data). The search will include all articles from their inception to February 2021. The search terms include

1. “low back pain” or “lumbago” or “lumbar disc herniation” or “lumbar sprain” or “backache” or “flatback syndrome” or “spinal instabilities”;
2. “Taiji” or “Tai Chi” or “Tai Chi Chuan” or “Qigong” or “Baduanjin” or “Wuqinxi” or “Yijinjing” or “Liuzijue”;
3. “psychological effects” or “anxiety” or “depression”. The search strategy details for PubMed are presented in Table 1.

**Table 1:** Search strategy (through PubMed).

Search Query	
#1	Search “ low back pain ”[tiab] OR “ lumbago ”[tiab] OR “ lumbar disc herniation ”[tiab] OR “ lumbar sprain ”[tiab] OR “ backache ”[tiab] OR “ flatback ”[tiab] OR “ spinal instabilities ”[tiab]
#2	“ Search ” Taiji ”[tiab] OR “ Tai Chi ”[tiab] OR “ Tai Chi Chuan ”[tiab] OR “ Qigong ”[tiab] OR “ Baduanjin ”[tiab] OR “ Wuqinxi ”[tiab] OR “ Yijinjing ”[tiab] OR “ Liuzijue ”[tiab]”
#3	Search “ psychological effects ”[tiab] OR “ anxiety ”[tiab] OR “ depression ”[tiab]
#4	Search “randomized clinical trial”[Article type]
#5	Search #1 AND #2 AND #3 AND #4

The similar terms will be translated into Chinese, and will be used in Chinese databases. In addition, the clinical registry will also be searched.

#### 2.2.2 Searching other resources

Relevant publications will be manually identified by searching the reference lists of related articles.

### 2.3 Study selection and data exaction

#### 2.3.1 Study selection

**Figure 1:**
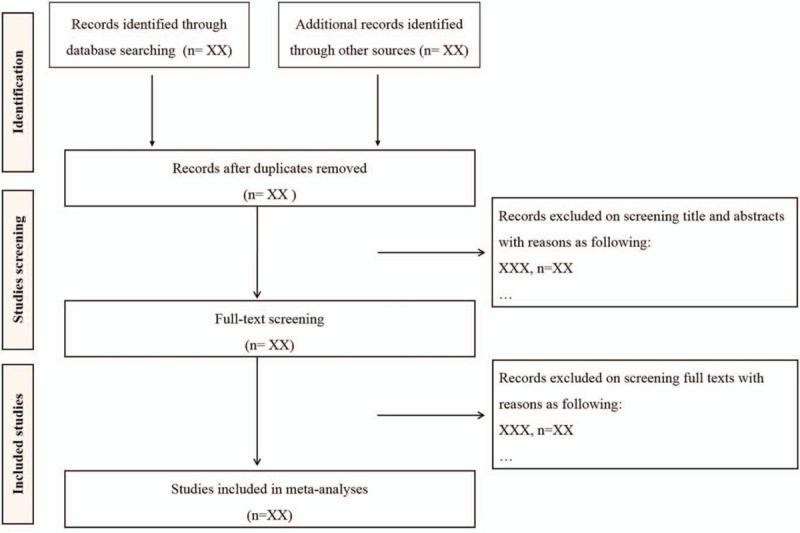
Flow chart of study selection.

The citation management software EndNote X9 will be used for literature selection. Two reviewers will independently select all literatures by assessing the titles and abstracts based on the predefined eligibility criteria. The full text of all relevant trials will be further evaluated for eligible studies. Any disagreements will be resolved through discussion between reviewers. The process and results of the studies selection will be presented in a flow chart with Figure 1.

#### 2.3.2 Data exaction

The data extraction will be performed by 2 reviewers independently. Data for collection include basic information (the first author, year of publication, and publication source, etc.), study characteristics (objectives of the study, method of randomization, method of blinding, method of analysis, etc), participant information (ample size, age, gender, ethnicity, and characteristics of LBP.), interventions (type of traditional Chinese mind-body exercises, control interventions, time of intervention, frequency of intervention, duration of a sessions, follow-up time, etc), and outcomes (the primary outcomes are focused on psychological effects including depression and anxiety; second outcomes include pain, disability, and quality of life). Any discrepancies will be resolved by discussion between reviewers.

### 2.4 Quality assessment

The quality of the included studies will be independently conducted by 2 reviewers using the Physiotherapy Evidence Database scale. Risk of bias of eligible studies will be assessed by Cochrane tool with risk of bias by 2 reviewers, which includes 7 items: random sequence generation, allocation concealment, blinding of patients, blinding of testers, blinding of outcome evaluators, outcome data incompletion, and selective reporting. The results of the assessment will be divided into low risk, unclear, and high risk. The overall quality of evidence will be assessed using the Grades of Recommendation, Assessment, Development and Evaluation framework including the risk of bias, inconsistency, indirectness, imprecision, and publications bias. Any disagreement will be resolved through discussion.

### 2.5 Data synthesis and analysis

The meta-analysis will be conducted using Review Manager Version 5.3 software. Continuous data will be presented as the mean difference and standardized mean difference with 95% confidence intervals. The test of *I*^*2*^ will be used to identify the heterogeneity: low level (*I*^*2*^ values of 25%–50%), moderate level (*I*^*2*^ values of 50%–75%), and high level (*I*^*2*^ values more than 75%). This review will use fixed-effect models if the *I*^2^ test will be not significant (*P* value less than .1), but random effect models will be significantly different. The subgroup analysis also will be conducted based on different types of traditional Chinese mind-body exercises, different intervention time, and different outcomes, if there are more than 3 eligible studies.

Sensitivity analysis will be used to assess the reliability of the combined results of meta-analysis, which combine the effect size after eliminating each of the included studies, or after changing the inclusion or exclusion criteria or eliminating types of studies. An Egger test was performed to examine publication bias, and publication bias will be determined from a corresponding *P* value less than .05.^[11]^ If relevant data are not reported, the corresponding authors will be contacted to get detailed information. If the meta-analysis is not possible, a narrative synthesis of the available data will be conducted.

## 3 Discussion

The current systematic review will conduct a comprehensive literature search to assess the psychological effects of traditional Chinese mind-body exercises in the management of LBP. Both electronic databases and grey literature will be searched for avoiding missing any potential eligible studies. The primary outcomes will focus on anxiety and depression of patients with LBP. The detailed subgroup analysis also will be conducted based on different types of traditional Chinese mind-body exercises, different intervention time, and different outcomes. The results of this meta-analysis will summarize the latest evidence on assessing the psychological effects of traditional Chinese mind-body exercises for LBP, which may benefit both clinicians and patients with chronic LBP.

However, there are some potential limitations in the current review. For example, there are different Taiji schools in China. So different Taiji may result in significant heterogeneity. The level of methodological quality of potential eligible studies also should be focused. In addition, limited to language ability, the documents only be in English and Chinese, and the studies in other languages may be ignored.

## Author contributions

**Conceptualization:** Qian Huang, Xiaonian Lu, Tianxiang He, Juan Du, Peiguo Zhang.

**Funding acquisition:** Qian Huang, Peiguo Zhang.

**Methodology:** Tianxiang He, Juan Du, Peiguo Zhang.

**Project administration:** Xiaonian Lu, Tianxiang He, Juan Du.

**Writing – original draft:** Qian Huang, Tianxiang He, Juan Du.

**Writing – review & editing:** Xiaonian Lu, Tianxiang He, Peiguo Zhang.
